# 

**Published:** 1889-04-27

**Authors:** 


### Amusements and Relaxation.

**Word Competition.**

Commenced April 6th, Ends June 29th.

Three Prizes of 15s., 10s., 5s., will be given for the largest number of words derived from the words set for dissection.

Proper names, abbreviations, foreign words, words of less than four letters, and repetitions are barred ; plurals, and past and present participles of verbs, are allowed. Nuttall's *Standard* dictionary only to be used.

N.B.—All words sent in must be arranged *alphabetically*, with correct total affixed.

The word for dissection for this, the *Fourth*, week of the quarter being

"BALFOUR."

**Results of Second Week.**

| Names. | April 18. | Totals. | Names. | April 18. | Totals. |
|---|---|---|---|---|---|
| Patience | 64 | 237 | See-saw | 50 | 183 |
| Nurse Duty | 59 | 231 | Rosemont | 42 | 173 |
| Lightowlers | 61 | 233 | Jenny Wren | — | 126 |
| Scrooge | 63 | 235 | Eric | 53 | 175 |
| Reldas | 61 | 232 | Tiny | 51 | 171 |
| Tinie | 63 | 233 | N'importe Qui | 54 | 171 |
| K. G. | 52 | 221 | Pollie (Green) | 29 | 141 |
| Broxbourne | 49 | 218 | W. G. R. | 33 | 142 |
| Lauriston | 60 | 225 | Robe | 43 | 150 |
| Spes | 55 | 220 | Beryl | 34 | 141 |
| Gretta | 62 | 224 | Nesta | — | 91 |
| Francesca | 57 | 215 | Daffodil | — | 89 |
| Heatherbell | 36 | 185 | A Schoolgirl | 36 | 117 |
| Sycamore | — | 149 | Montague | — | 79 |
| Arctus | 54 | 199 | Sheeny | — | 78 |
| Amicus | 57 | 201 | Alice R. | — | 63 |
| Scratcher | — | 143 | Belfast | 58 | 58 |
| Jemima | — | 138 | Gentian | 53 | 53 |
| Concours | 36 | 177 | Hope | 39 | 39 |
| C. J. S. | — | 134 | | | |

**Notice to all Correspondents.**

N.B.—All letters referring to this page which do not arrive at 139 and 140, Salisbury-court, London, E.C., by the *first post on Thursdays*, and are not addressed PRIZE EDITOR, will in future be disqualified and disregarded.

The following vacancies are announced this week, the amount of the salary in each case being given after the name of the institution :

**Resident Medical Officers.**

Birmingham General Dispensary. Resident Surgeon.—£150 per annum.

Brighton, Hove, and Preston Dispensary. Two House Surgeons.—£140 per annum.

Clinical Hospital for Women and Children, Manchester. House Surgeon.—£80 per annum, with board, &c.

Crichton Royal Institution, Dumfries. Junior Medical Assistant.—£100 per annum.

Denbighshire Infirmary, Denbigh. House Surgeon.—£85 per annum, with board.

Grove Hall Asylum, Bow, E. Assistant Medical Officer. £120 per annum, with board, &c.

Montrose Royal Lunatic Asylum. Junior Assistant Medical Officer.—£100 per annum, with board, &c.

Public Dispensary, Clare Market. Resident Medical Officer.—£105 per annum, with board, &c.

Royal Isle of Wight Infirmary, Ryde. House Surgeon and Secretary.—£50 per annum, with board, &c.

Smedley's Hydropathic Establishment, Matlock. Resident Junior Physician.—£100 per annum, with board, &c.

Staffordshire County Lunatic Asylum, Stafford. Junior Assistant Medical Officer. — £100 per annum, with board, &c.

Torbay Hospital and Provident Dispensary, Torquay. House Surgeon and Dispenser.—£80 per annum, with board, &c.

Western General Dispensary, Marylebone-road. Junior House Surgeon.—£75 per annum.

**Non-Resident Medical Officers.**

Lampeter Union. Medical Officer and Public Vaccinator. £40 per annum, with fees.

Middlesex Hospital, W. Medical Registrar.

May 8

0

### THE ROYAL NUMBER

OF

### The Hospital

CONTAINING AN ACCOUNT OF THE PUBLIC LIFE OF THEIR ROYAL HIGHNESSES THE

### Prince & Princess of Wales

AND ITS EFFECTS UPON THE SOCIAL AND PHILANTHROPIC LIFE OF OUR OWN TIMES.

No. 135, Vol. VI.] APRIL 27, 1889. [PRICE ONE PENNY.

**Patron: The QUEEN.**

### NATIONAL HOSPITAL FOR THE PARALYSED AND EPILEPTIC

(ALBANY MEMORIAL),

**QUEEN SQUARE, BLOOMSBURY.**

The LORD ARCHBISHOP of CANTERBURY has consented to preside at a FESTIVAL DINNER to take place at the Hôtel Métropole, on THURSDAY, 23rd May next, when his Grace will be supported by the Right Hon. the Lord Chancellor and others.

The National Hospital was founded in public meeting at the Mansion House in 1859.

In 1885 a new and suitable building was **opened by H.R.H the Prince of Wales, accompanied by H.R.H. the Princess of Wales and other members of the Royal Family.** With Her Majesty's gracious permission, a chief portion was constituted a **Memorial to H.R.H. the late Duke of Albany.**

The Hospital contains 175 beds, and the attendances of out-patients in 1888 exceeded 23,000. The Hospital receives patients from every part of the kingdom. There are special Wards for middle-class patients in straitened circumstances who contribute 21s. per week. Provision by way of Life Pensions is made for 75 incurable sufferers.

Upon the results of the Festival must depend, in a great measure, the ability to keep all the Wards open. The Board therefore earnestly plead for such assistance and co-operation as will prevent any curtailment of the beneficent work carried on.

The names of gentlemen who will undertake the office of Steward, and contributions to the Fund, will be thankfully received. Dinner tickets, ladies or gentlemen, 21s. each.

(9) B. BURFORD RAWLINGS, Secretary-Director.

### The British Home for Incurables was the first Charity in England
patronized by

### H.R.H. THE PRINCESS OF WALES.

**Office: 73, CHEAPSIDE, E.C.** **R. G. SALMOND, Secretary.** (25)

